# How confident are general dental practitioners in their decision to administer antibiotic prophylaxis? A questionnaire study

**DOI:** 10.1186/1472-6947-8-57

**Published:** 2008-12-08

**Authors:** Eva Ellervall, Berndt Brehmer, Kerstin Knutsson

**Affiliations:** 1 Faculty of Odontology, Malmö University, Malmö, Sweden; 2 Department of War Sciences, Swedish National Defence College, Stockholm, Sweden

## Abstract

**Background:**

Common dental procedures induce bacteremia. To prevent infectious complications from bacteremia in patients with specific medical conditions, antibiotic prophylaxis is considered. Recommendations are often unclear and ambiguous. In a previous study we reported wide variations in general dental practitioners' (GDPs') administrations of antibiotic prophylaxis. We hypothesized that within such a conflicting clinical area, decisions are made with a high level of personal uncertainty. This study examined GDPs' confidence in their decisions and analyzed the extent to which case-related factors might explain individual variations in confidence.

**Methods:**

Postal questionnaires in combination with telephone interviews were used. The response rate was 51% (101/200). There were no significant differences between respondents and non-respondents regarding sex, age, or place of work. The GDPs were presented to patient cases of different medical conditions, where some should receive antibiotic prophylaxis according to recommendations when performing dental procedures that could cause gingival bleeding. The GDPs assessed on visual analogue scales how confident they were in their decisions. The extent to which case-related factors, medical condition and dental procedure, could explain individual variation in confidence was analyzed.

**Results:**

Overall the GDPs exhibited high confidence in their decisions regardless of whether they administered antibiotic prophylaxis or not, or whether their decisions were in accordance with recommendations or not. The case-related factors could explain between 30–100% of the individual variation in GDPs' confidence. For 46%, the medical condition significantly explained the individual variation in confidence. However, for most of these GDPs, lower confidence was not presented for conditions where recommendations are unclear and higher confidence was not presented for conditions where recommendations are more clear. For 8% the dental procedure significantly explained the variation, although all procedures could cause bacteremia. For 46% neither the medical condition nor the dental procedure could significantly explain the individual variation in confidence.

**Conclusion:**

The GDPs presented high confidence in their decisions, and the majority of GDPs did not present what could be considered a justified varied level of confidence according to the clarity of recommendations. Clinicians who are overconfident in their decisions may be less susceptible to modifications of their behavior to more evidence-based strategies.

## Background

Common dental procedures induce transient bacteremia. To prevent infectious complications from transient bacteremia in patients with specific medical conditions, antibiotic prophylaxis is considered. Decisions on antibiotic administration should weigh the risk of bacteremia inducing complications against the risk of adverse reactions to antibiotics and the risk of antibiotic resistance [1]. Studies have reported wide variations in general dental practitioners' (GDPs') administration strategies of antibiotic prophylaxis [2,3]. Within medical and oral health care there are wide variations in clinicians' decisions about treatment [4]. Further, the constant flow of information and technologies being developed makes it reasonable to assume that variations in care will continue to increase [5].

Medical uncertainty contributes to the significant variability in clinical practice [4]. Uncertainty could be divided into three different types [6]. The first results from clinicians having incomplete knowledge of the situation. The second is due to limitations of present medical knowledge. The third is a combination of the first two, where there is difficulty distinguishing between personal lack of knowledge and limitations in current knowledge [6]. Within this clinical area there is lack of evidence for the effectiveness of antibiotic prophylaxis [7,8], which could affect clinicians' personal confidence in their decisions.

Even though many guidelines for the rational use of antibiotic prophylaxis have been published, recommendations are often unclear and ambiguous [9]. In a previous study, we reported wide variations in GDPs' administration strategies of antibiotic prophylaxis [2]. For medical conditions where recommendations are unclear, for example not well-controlled diabetes and kidney transplant, the GDPs varied in their administration strategies. However the GDPs also varied in their decisions for medical conditions where recommendations are more clear, for example heart valve prosthesis [2]. Even though large variations in treatment strategies exist, it has been reported that the majority of clinicians believe that their colleagues would make similar decisions as themselves, thus assuming the existence of broad consensus [10,11]. Obviously there seem to be an opposition between the real situation and the clinicians' understanding of it. Dentists' assessments of indications for treatment options have been studied, i.e. how strong they judge the indication is to perform a certain treatment [10,12-14]. But to our knowledge, no previous studies have been published that present dentists' confidence in their treatment decisions.

There are a number of theories on human cognitive processes or mental models. One model is the Social Judgement Theory (SJT). This model focuses on the actual decision made in relation to a well-defined task requiring judgement and on how the judges (i.e. the GDPs) use the available information – "cues" (i.e. medical condition and dental procedure) – to reach that decision [15]. Our aim was to examine, with the use of the SJT, the confidence of GDPs in their decisions on administration of antibiotic prophylaxis to patients with different medical conditions and to analyze the extent to which case-related factors, medical condition and dental procedure, might explain individual variation in confidence.

Within such a conflicting clinical area, with wide variation in GDPs' administration strategies for different medical conditions and dental procedures [2] and where recommendations are unclear [16,17], the following hypothesis guided the design of the study:

• Decisions are made with a high level of personal uncertainty and therefore GDPs will present low confidence in their decisions (values below 30 mm on a visual analogue scale was considered as low confidence, and values above 70 mm was considered as high confidence).

• No significant differences in confidence assessments will be found between men and women, between GDPs working in Public Dental Service and private dental service, between ages or between GDPs with varying years of professional experience. This hypothesis was based on results from studies where no differences in judgements were found by clinicians with varying years of professional experience [10,11].

• Since recommendations are unclear for many medical conditions, the individual variation in confidence will largely be explained by the medical condition. All the included dental procedures could directly or indirectly cause gingival bleeding, which would indicate that if antibiotic administration is considered for one procedure it should also be considered for the other procedures and thus the confidence should be equal for all the procedures. However, our earlier study presented that GDPs differed in their decisions depending on which procedure they performed. Therefore, we assumed that the dental procedures would also explain the individual variation in confidence.

## Methods

### Setting and participants

In a computer-generated randomization procedure, 200 GDPs from two regions in Sweden were selected to participate in the study. The response rate was 51% (101/200). The share of male respondents was 57% and of female respondents 43%. These distributions reflect the distributions of female and male dentists in the membership register of the Swedish Dental Association. The mean age of the respondents was 48 years (range 26–64 years). The mean number of years of professional experience as GDPs was 20 years (range 1–44 years). More respondents worked in the Public Dental Service (60%) than in private dental service (40%).

There were no significant differences between respondents and non-respondents regarding sex, age, or place of work (public/private dental service) (P > 0.05), analyzed with the chi-square test. Thus, the group of respondents could be considered representative of the initial sample of GDPs who had been randomly selected for participation.

### Data collection procedure and variables assessed

A postal questionnaire in combination with a structured telephone interview was used. Informed consent was obtained from all participants. Initially, an inquiry was sent to the GDPs asking whether they were willing to participate in the study. The inquiry included an introductory letter, a document of consent to participate, and a reply-paid envelope. Two reminders were sent to non-responding GDPs. We also applied other steps that are described in guidelines on how to improve response rates to postal questionnaires, for example using a short questionnaire to enhance the likelihood of receiving more responses [18]. The present study is the second part of a more extensive questionnaire study on administration strategies of antibiotic prophylaxis by GDPs. Data were collected between January and June 2003. The Ethics Committee at Lund University in Sweden approved the study (LU 305-02).

The questionnaire comprised eight simulated cases of patients with different medical conditions. The questionnaire was tested by two GDPs and modified (clarifying questions and extended with one case) before the final version was developed. These were the medical conditions:

1. Type 1 diabetes mellitus, insulin-dependent, well controlled.

2. Type 2 diabetes mellitus, medicating with oral anti-diabetic agents, well controlled.

3. Type 1 diabetes mellitus, insulin-dependent, not well controlled.

4. Moderate hypertension, medicating with beta-receptor antagonist.

5. Myocardial infarction 3 months ago, medicating with ACE inhibitor, beta-receptor antagonist, low-dose aspirin, and simvastatin.

6. Kidney transplant 3 years ago, medicating with immunosuppressive and beta-receptor antagonist for moderate hypertension, well controlled without complications.

7. Heart valve prosthesis, medicating with warfarin.

8. Hip prosthesis, replacement performed 3 years ago.

For each medical condition, three types of dental procedures were presented:

A. Scaling lingually in the lower jaw (probing pocket depth between 2 and 3 mm).

B. Surgery, for example, removal of an asymptomatic tooth.

C. Root canal treatment due to pulp exposure as a result of caries (the pulp is vital).

These dental procedures were selected to represent interventions that could produce gingival bleeding. Root canal treatment (dental procedure C) *per se *is not generally a procedure that is considered to cause gingival bleeding and require antibiotic prophylaxis. But placement of rubber dam clamps may cause gingival bleeding and thus generate bacteremia [19].

**Figure 1:**
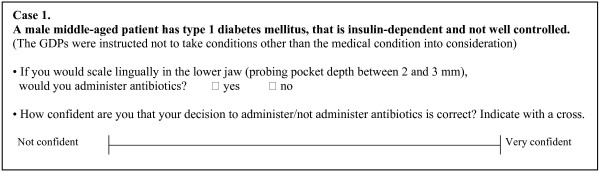
One of the cases presented to the GDPs.

For each case, the GDPs were asked to consider the questions presented in Figure 1. The medical condition and the dental procedure were the "cues", i.e. the information in each case that we analyzed. Other information in the case presentations, for example age, was constant.

There is lack of evidence for administrating antibiotic prophylaxis [7,8]. However, recommendations exist and are often based on consensus and not on evidence. According to our interpretation of local recommendations [16,17] the GDPs would be expected to administer antibiotic prophylaxis to patients with not well-controlled diabetes, kidney transplant, and heart valve prosthesis. They could be expected to administer antibiotics for all three procedures, since they all could cause gingival bleeding.

### Data analysis

Each GDP's assessment of confidence in a decision was measured to the nearest millimetre on a visual analogue scale (VAS) where 0 mm represented the end-point "not confident" and 100 mm the end-point "very confident".

Differences in confidence assessments between GDPs who would administer antibiotic prophylaxis and GDPs who would not, were analyzed with Independent Samples t-test (P = 0.05). Differences in confidence assessments between men and women, between GDPs working in Public Dental Service and private dental service, between ages and between GDPs with varying years of professional experience was analyzed using a multiple linear regression.

For each GDP, we calculated an R^2^-value presenting the extent to which variation in GDPs' confidence assessments could be explained by the factors medical condition and dental procedure (two-way ANOVA analysis). In the R^2^-analysis, we also evaluated whether the factors significantly explained each GDPs' variation in confidence. Based on which of the factors that significantly explained the GDPs' variation in confidence we organized the GDPs into different classifications.

## Results

Table 1 presents GDPs' administration strategies of antibiotic prophylaxis and their assessments of confidence, according to GDPs who would administer antibiotics and GDPs who would not. The overall mean in confidence assessments for the entire sample of GDPs was 79 mm on the VAS and the range was 54–93 mm. Generally, the GDPs presented high confidence in all their decisions regardless of whether they administered antibiotic prophylaxis or not (P > 0.05). There were a few exceptions. In both cases with patients with well-controlled diabetes, GDPs who would not administer antibiotics were more confident than GDPs who would administer antibiotics for the procedure of tooth removal (P < 0.05). In the patient with not well-controlled diabetes and the patient with an episode of myocardial infarction, GDPs who would not administer antibiotics were more confident than GDPs who would administer antibiotics for the procedure of root canal treatment (P < 0.05).

**Table 1:** GDPs' (*n *= 101) administration strategies and their assessments of confidence

Medical condition	Dental procedure	Administer antibiotics			Confidence (mean)	
		Yes	No	Total	Yes	No
Type 1 diabetes, well-controlled	Scaling	-	101	101	-	^c^92
	Tooth removal	10	91	101	60	^b^89
	Root canal treatment	1	100	101	78	^c^93
Type 2 diabetes, well-controlled	Scaling	-	101	101	-	^c^92
	Tooth removal	6	95	101	57	^b^89
	Root canal treatment	-	101	101	-	^c^92
Type 1 diabetes, not well-controlled	Scaling	30	71	101	77	80
	Tooth removal	77	24	101	80	72
	Root canal treatment	22	79	101	68	^b^78
Moderate hypertension	Scaling	-	101	101	-	^c^91
	Tooth removal	1	100	101	54	^c^90
	Root canal treatment	-	101	101	-	^c^92
Myocardial infarction	Scaling	28	73	101	76	80
	Tooth removal	54	47	101	76	79
	Root canal treatment	24	77	101	69	^b^81
Kidney transplant	Scaling	50	46	^a^96	72	78
	Tooth removal	83	11	^a^94	82	73
	Root canal treatment	39	56	^a^95	72	73
Heart valve prosthesis	Scaling	75	25	^a^100	86	85
	Tooth removal	97	1	^a^98	91	^c^68
	Root canal treatment	63	37	^a^100	80	80
Hip prosthesis, 3 years ago	Scaling	10	91	101	77	84
	Tooth removal	41	60	101	73	81
	Root canal treatment	12	89	101	75	84

The GDPs assessed their confidence on visual analogue scales (VAS), where 0 mm represented the end-point "not confident" and 100 mm the end-point "very confident". Measurements were made to the nearest millimetre.

^a ^= A few GDPs answered "would contact patients' physician".

^b ^= GDPs who would not administer antibiotics were more confident compared to GDPs who would administer antibiotics (P < 0.05).

^c ^= No statistical comparison was possible since there were no or too few GDPs in the yes or no groups.

There were no significant differences in confidence assessments between men and women, between GDPs working in Public Dental Service and private dental service, between ages or between GDPs with varying years of professional experience (P > 0.05).

The individual variation in GDP's assessments of confidence explained by the medical condition and dental procedure (R^2^) varied between 0.293–0.996 (Table 2). Based on which factors that significantly explained individual variation in confidence, the GDPs were organized into three different classifications:

**Table 2:** GDPs' individual variation in their confidence assessments, explained by the factors medical condition and dental procedure (R^2^)

GDP	R^2^	Medical condition	Dental procedure	GDP	R^2^	Medical condition	Dental procedure
1	0.407			52	0.700		
2	0.398			53	0.598		
3	0.798	*		54	0.419		
4	0.450			55	0.973	*	
5	0.689		*	56	0.783	*	
6	0.691	*		57	0.669		*
7	0.569			58	0.632	*	
8	0.758	*		59	0.491		
9	0.714		*	60	0.337		
10	0.394			61	0.732	*	
11	0.942	*		62	0.538		
12	0.910	*		63	0.807	*	
13	0.527			64	0.632		
14	0.687	*		65	0.472		
15	0.506			66	0.735	*	
16	0.794	*		67	0.955	*	
17	0.757	*		68	0.688		*
18	0.445			69	0.459		
19	0.307			70	0.444		
20	0.812	*		71	0.386		
21	0.611		*	72	0.747		*
22	0.824	*		73	0.729		*
23	0.586			74	0.600		
24	0.626			75	0.645	*	
25	0.809	*		76	0.613	*	
26	0.373			77	0.725	*	
27	0.391			78	0.864	*	
28	0.741	*		79	0.480		
29	0.381			80	0.677	*	
30	0.996	*		81	0.293		
31	0.350			82	0.863	*	
32	0.638	*		83	0.607		
33	0.605			84	0.316		
34	0.478			85	0.548		
35	0.402			86	0.610	*	
36	0.739	*		87	0.375		
37	0.691	*		88	0.607	*	
38	0.772	*		89	0.985	*	
39	0.599		*	90	0.655	*	
40	0.559			91	0.732	*	
41	0.357			92	0.635	*	
42	0.975	*		93	0.589	*	
43	0.443			94	0.481		
44	0.842	*		95	0.888	*	
45	0.565			96	0.366		
46	0.502			97	0.654	*	
47	0.587			98	0.560		
48	0.387			99	0.744	*	
49	0.930	*		100	0.739	*	
50	0.483			101	0.870	*	
51	0.742	*					

* P < 0.05

• For 46 of the GDPs (~45%), the medical condition explained the individual variation in confidence (P < 0.05) (R^2 ^0.589–0.996). However only 7 of the GDPs (~15%) presented what could be considered a justified varied level of confidence, i.e. lower confidence for conditions where recommendations were unclear and higher confidence for conditions where recommendations were more clear.

• For 8 of the GDPs (~8%), the dental procedure explained the variation (P < 0.05) (R^2 ^0.599–0.747). Nearly all the GDPs administered antibiotics for the procedure of tooth removal. Their confidence in the decision for tooth removal was lower than for scaling and root canal treatment, although all three procedures could cause bacteremia.

• For 47 of the GDPs (~47%), neither the medical condition nor the dental procedure explained the variation (P > 0.05) (R^2 ^0.293–0.700).

## Discussion

### Methodological considerations

The 51% response rate in our study can be compared to response rates of 20–60% reported in similar studies [3,9,20]. One reason for the rather low response rate in this study could be that the method of collecting answers, a questionnaire and a telephone interview, was considered time-consuming for the respondents. But the sample could be considered representative for the GDPs who were randomly selected to be included in this study, since there were no differences between respondents and non-respondents regarding sex, age, or place of work.

The GDPs made their decisions about paper cases instead of actual patients. Although the use of paper cases have been criticised [21], the method is practical and has been validated [22,23]. To make cases realistic and vivid to respondents, it is important to include details in the presentation of the patients [24]. In our case presentations, we deliberately narrowed the information to focus on the medical condition and the dental procedure. The reason for this was that we were only interested in the clinicians' decision-making process, elucidating their knowledge when administrating antibiotic prophylaxis and thus we wanted to exclude the "noise" from patients' preferences and other information. Furthermore, the medical condition and the dental procedure is also the information that recommendations are based upon. However, we acknowledge that our presentation of the cases was not very vivid and this is a limitation of our study.

The GDPs were asked to express their confidence in their decisions concerning each of the medical conditions and dental procedures using the VAS. The VAS has been reported as an appropriate method for measuring GDPs' and oral surgeons' assessments of the strength of the indication to remove third molars, since it has a high reliability. The mean correlation coefficient of intra-examiner reliability was 0.72 for the GDPs and 0.84 the for oral surgeons [25]. When we asked the GDPs "How confident are you that your decision to administer/not administer antibiotics is correct?" (see question in Figure 1), we did not mean correctness in relation to recommendations or evidence. We meant the GDP's own personal viewpoint of correctness related to each case. In the telephone interviews, we tried to ensure that our intention was understood. However, we can not be certain that this was accomplished since respondents' interpretation of questions varies [26]. Further, the study was descriptive and we did not examine the GDPs' cognitive process since we did not ask them to vocalise their thoughts when they assessed their confidence on the VAS.

### Considerations of the results

The results from our study showed that GDPs presented an overall high confidence in their decisions, regardless of whether they chose to administer antibiotics or not, or whether their decisions were according to recommendations or not. Thus our first hypothesis, that GDPs will present low confidence in their decisions, could be rejected. Studies on clinicians' confidence in their judgements and decisions are sparse. In judgements on diagnosis, results show that clinicians' are generally very confident that their diagnoses are correct although they are often inaccurate [27]. In treatment decisions, clinicians presented high confidence although they varied in their decisions and no consensus existed on which decision was optimal [28]. These results are confirmed by our findings.

The GDPs' high confidence could be questioned since evidence for the administration of antibiotic prophylaxis is inexplicit for many of the medical conditions discussed in this study [7,8]. If translated into "real-life situations", high confidence could be explained by GDPs who wish to avoid acknowledging uncertainty in their decisions, because it might increase patients' anxiety and could affect the relationship between the clinician and the patient [29]. It might also be less time-consuming to administer antibiotics in cases where there is doubt instead of discussing or trying to persuade the patient. Many patients probably feel that they are being cared for when they receive a concrete intervention, whose purpose is to prevent complications. By doing an intervention that diminishes their uncertainty and satisfies the patient, the GDPs justify their high confidence assessments. Reports of incorrect treatment to the Swedish National Board of Health and Welfare are seldom made by patients because of over-use of an intervention, but rather concerning shortcomings of interventions. Furthermore, the results from this study agree with the theory of "professional certainty", which states that clinicians are very certain/confident that their practice is correct, irrespective of how much it differs from that of others [30].

Although the GDPs generally presented high confidence in their decisions, there were a few exceptions where GDPs who would not administer antibiotics were more confident than GDPs who would. These results were revealed for the two patients with well controlled diabetes and the patient with an episode of myocardial infarction. Approximately 300 000 people in Sweden have diabetes [31] and 587 000 people have had an episode of myocardial infarction between 1987–2005 [32]. Based on these figures, all GDPs are familiar with these patients in their practice. GDPs that were more confident in their decisions might have reflected on their practice for these patients and chose not to administer unnecessary antibiotic prophylaxis. Perhaps, GDPs that were less confident in their decision lacked knowledge that such patients would not benefit from antibiotic prophylaxis.

We found no significant differences in confidence assessments when analyzing the background variables (sex, age, years of professional experience, and place of work). This could imply that other characteristics, perhaps personality, could explain the GDPs' individual variation in confidence.

Our second hypothesis, assuming that the medical condition would largely explain GDPs' variation in confidence but also that the dental procedure would explain variation in confidence, could be accepted. The case-related factors could explain between 30–100% of the individual variation in GDPs' confidence. For some of the GDPs, the medical condition significantly explained the variation in confidence. It could be expected that the varied level of confidence for these GDPs was jusified if they assessed lower confidence for conditions where recommendations are unclear and higher confidence for conditions where recommendations are more clear. However, for only 15% of these GDPs the variation in confidence followed that principle.

For other GDPs, the dental procedure significantly explained the variation. These GDPs almost only administered antibiotics for the procedure of tooth removal. Their confidence in the decision for tooth removal was lower than for scaling and root canal treatment. Perhaps the GDPs were unaware or uncertain of the fact that bacteremia occurs when gingival bleeding is present, independent of the severity of the procedure [19]. So, although they lacked confidence in this decision they preferred to be on the safe side and therefore chose to administer antibiotics for the procedure of tooth removal, which is the most invasive procedure of the three.

Finally, for some GDPs neither the medical condition nor the dental procedure significantly explained the variation in confidence. These GDPs could be considered inconsistent. But that does not mean that they did not rely on any of the factors, even though they did not do so in a significant way.

Our third hypothesis, that there would be no differences in confidence between men and women, between GDPs working in Public Dental Service and private dental service, between ages or between GDPs with varying years of professional experience, was confirmed. To be able to grasp more personal characteristics, such as reasons and processes behind GDPs' behaviors, in-depth interviews should be performed to collect qualitative data [33]. Still, our results presenting an overall high confidence in GDPs' administration strategies of antibiotic prophylaxis is surprising. Generally no consideration is taken, as far as we could explore, to concerns that evidence is lacking or that recommendations are unclear in their expressed confidence.

There has been a public discussion in this field and recommendations have recently changed [34-36]. It is logic to assume that this would make GDPs confused and could impact the GDPs' current confidence in their decisions. However, since this study revealed an overall high confidence among the GDPs regardless of whether their decisions were in accordance with recommendations or not, we are not convinced that the changes will influence GDPs' confidence in their decisions.

## Conclusion

The GDPs presented high confidence in their decisions, regardless of whether or not they chose to administer antibiotics, or whether their decisions were according to recommendations or not. The case-related factors (medical condition and dental procedure) could explain between 30–100% of the individual variation in GDPs' confidence. However only 7 of all the GDPs (~15%) presented what could be considered a justified varied level of confidence, i.e. lower confidence for conditions where recommendations were unclear and higher confidence for conditions where recommendations were more clear. Clinicians who are overconfident that their decision is correct may be less susceptible to modifications of their behavior to more evidence-based strategies [37]. Knowledge about the processes of human change is limited [38]. Research on clinicians' beliefs, attitudes, and judgements is therefore needed to discover how successful interventions can be implemented. This research must also take into account that health care delivery is becoming increasingly complex [38].

## Competing interests

The authors declare that they have no competing interests.

## Authors' contributions

EE collected and analyzed the data, and wrote the manuscript. BB was a consultant during the study and was involved in the analyses and interpretation of the results. KK was supervisor, and contributed to the analyses and writing. All authors read and approved the final manuscript.

## Pre-publication history

The pre-publication history for this paper can be accessed here:


